# Treatment of Diphtheria

**Published:** 1868-05-15

**Authors:** DeLaskie Miller

**Affiliations:** Professor of Obstetrics, and Diseases of Women and Children, in Rush Medical College


### TREATMENT OF DIPHTHERIA.

*Submitted to the Chicago Medical Society by* DeLaskie Miller, M.D., *Professor of Obstetrics, and Diseases of Women and Children, in Rush Medical College.*

Mr. President:—The subject before the Society for discussion this evening being the *Treatment* of Diphtheria, reference to the history, symptoms, and diagnosis of the disease is precluded.

This circumstance, however, presents but few embarrassments in this discussion. We must accept the diagnosis furnished us, and in general terms, also the pathology. Fortunately the materials are abundant, both in the literature of the profession upon this subject, and in the personal observations of the members of this Society. The repeated visitations of diphtheria to this city during the last ten years, have afforded ample opportunities to verify or correct the descriptions of this disease which have reached us from other sections of the country.

To be rational and successful, the treatment of any disease must be based on definite and correct ideas of its pathology. The views entertained and promulgated, of the nature of diphtheria on its first appearance, abroad as well as on this side of the Atlantic, were in the main partial and defective, in this, that the disease was regarded as essentially an *Angina* — a local disease of the throat. And believing this, efforts were directed mainly to the mitigation of local symptoms. From the time that Bretonneau named the disease, from what he believed to be an ever present and characteristic (?) appearance of the throat, writers have given the first and most prominent place to a description of the appearances in and about the throat, and sometimes to the exclusion of the general symptoms; leading to but one conclusion, viz.: that the local manifestations were regarded by such as the disease. Bretonneau taught that "its specific character consists, anatomically, in the formation of a false membrane, of definite structure; pathologically, in the power of reproducing itself." And this doctrine has been reproduced, from first or second hand, again and again, from that time to this.

A few citations may satisfy that this position does no violence to fact. If we may infer what views are entertained of the nature of a disease by the course of treatment recommended for its cure, then we can be at no loss in the following instances, taken from many: Trousseau said, "Topical medication is, *par excellence*, the treatment, notwithstanding the opposition to it." West says, "Two main points are involved in the treatment of the disease; the one, the control of the local symptoms, the other the support of the constitutional powers;" and again, "local remedies then take a very prominent place in the treatment of diphtheria." Bennett advises "iced water and steam internally, and poultices externally." And Aitken, after diuretics and cathartics, inhalation of aqueous vapors, acidulated with *Acetic acid.* Condie seems to represent the opposite extreme by advising, 1st, Emetics; 2nd, Abstraction of blood; 3rd, *Calomel* in large and repeated doses. But these references must suffice.

Doubtless much remains to be learned in regard to the nature and treatment of diphtheria; sufficient, however, has been determined, to leave no doubt that its constitutional complications are even more important than the local manifestations, for if we subdue the former early, we prevent the latter.

In this connection the following inquiries are pertinent. In diphtheria, what danger is to be apprehended? What complication is to be feared? What sequelæ are to be warded off? The answers to these interrogatives will lead us to the positive indications of treatment.

1st. Prostration of the patient's strength, and exhaustion of the vital forces of the system. These are liable to appear early, and sometimes unexpectedly, The explanation is most readily found in the changes in the quality of the blood.

2nd. The local complications — swelling of the glands, and the formation of the false membrane, which may extend so as to endanger life.

3rd. Paralysis of certain parts, which in some cases aggravates the danger, and shows how profoundly the nervous centres may be implicated.

These possibilities should be kept constantly in mind, in the treatment of every case of diphtheria, and our most efficient agents applied to anticipate, and thus prevent them.

In almost every case, a cathartic is indicated, though the bowels may have moved at regular intervals, they are seldom thoroughly evacuated. The fæcal matter retained, besides causing local irritation, is constantly undergoing changes, and gives rise to the accumulation of fœtid gases, which are re-absorbed to produce their septic influences on the blood. This may be prevented by completely evacuating the bowels, not by a drastic purgative, which by its perturbing effect, might aggravate the depressing effects of the disease, but by a mild and efficient agent, such as *Ol. ricini*, with the *Ol. terebinthinæ*, in quantities and proportions to suit the age of the patient.

Commence immediately with a prescription containing anti-septic, tonic, restorative and eliminative agents. I am in the habit of prescribing something like the following, varying the proportions to meet the peculiarities of the particular case, viz.: ℞. *Tr. ferri Chloridi* ʒ ii, *Potas. chlorat.* ʒ ii., *Morph. muriat.* gr. i., *Acid. muriatic. dil.* ʒ ii., *Aq. destil.* ℥ i., *Syrupi*, ℥ ii. M. S. Dose, a teaspoonful every 2nd or 3rd hour; or in urgent cases, every hour.

It should be taken without further dilution, for in addition to its general effect, it is the most efficient local application we can make. To prevent irritation of the stomach, a few swallows of water may be taken just before the medicine. Nutritious food, liberally, is of the first importance. In the majority of cases no other treatment will be required.

I have followed this plan of treatment so long, and applied it in so many cases, with such almost unvarying success, that I feel justified in believing that these remedies may act upon the volume of the blood, and upon the capillary vessels, so promptly, as to prevent the exudation and the formation of false membrane upon the mucous surfaces. I believe we may prevent the appearance of albumen in the urine, or if it is already present, these remedies will lessen the quantity, and soon remove it. Of course I speak of the presence of albumen, as the result of diphtheria. May not the *Tinct. ferri chloridi* so sustain the innervation as to prevent the paralysis of the pharynx and the heart, which, in some cases, is the greatest element of danger? I believe so.

But we may be called after the membrane has formed; then if the remedies already prescribed do not arrest its further development, and especially should it be extending downward, as it sometimes does, I would hardly think of making local applications with the probang.

The objections to this mode of treatment are:

1st. That it is distressing to the patient.

2nd. If the false membrane has formed, and presents the hard, unyielding pellicle described by careful observers, the application is made to an insensitive surface, and can be beneficial only so far and to the extent that it acts as a chemical solvent.

3rd. It can seldom be applied to the entire surface implicated.

4th. Under such circumstances, the vital tissues involved are unaffected by the operation, because they are not touched by the application.

I believe that inhalations will fill the indications under these circumstances, more perfectly. When the spray is inhaled from the atomizer, we may charge it with agents which we may suppose produce an alterative effect upon the diseased structures, or a chemical action upon the deposit on the surface of the mucous membrane. Then we add to the effect of medicines, the soothing and relaxing effects of warm aqueous spray, carried into the passages as far as the disease extends.

*Hydrochloric acid* has the power of dissolving fibrin, a weak solution, no stronger than 1 part in 1000 will do this. Should this not be used by the atomizer?

Or upon the supposition that *sporules* or *fungi* constitute an essential part of the false membrane, as has been affirmed, would not the spray of a weak solution of *Carbolic acid* be the most active and certain agent for their destruction, at our command? From the reputed efficacy of *Carbolic acid* in destroying parasites of all kinds, should we not expect beneficial results from its use in this manner?

I speak interrogatively of this use of these remedies. I have not been compelled to use them. Analogically they are indicated.

I make no reference to tracheotomy, for I have had no experience that would warrant me in consuming the valuable time of the Society. Nor have I any *special* suggestions to make upon the treatment of paralysis, which is an occasional sequela of diphtheria.

A desire to abbreviate, as much as possible, what I had to say, has prevented the elaboration of several points, which come within the scope of discussion, to the extent their importance demands. Such as the use of stimulants, and the mode of introducing nourishment in extreme cases; the management of enlargement of the glands, sub-maxillary and others, so frequently met with, which is caused by engorgement, and, fortunately, unattended by a tendency to suppurate.

